# Learning the representation of instrument images in laparoscopy videos

**DOI:** 10.1049/htl.2019.0077

**Published:** 2019-11-26

**Authors:** Sabrina Kletz, Klaus Schoeffmann, Heinrich Husslein

**Affiliations:** 1 Institute of Information Technology, Klagenfurt University, Austria; 2 Department of Gynecology and Obstetrics, Medical University of Vienna, Austria

**Keywords:** object detection, video signal processing, learning (artificial intelligence), image classification, gynaecology, surgery, image sequences, convolutional neural nets, image motion analysis, medical image processing, image representation, laparoscopy videos, automatic recognition, recognition approaches, instrument frames, video frames, classification tasks, action recognition, noninstrument images, learned activation patterns, instrument count classifications, transfer learning, adverse event analysis, binary classification, convolutional neural network, GoogLeNet, cholecystectomy, gynaecology, instrument images representation

## Abstract

Automatic recognition of instruments in laparoscopy videos poses many challenges that need to be addressed, like identifying multiple instruments appearing in various representations and in different lighting conditions, which in turn may be occluded by other instruments, tissue, blood, or smoke. Considering these challenges, it may be beneficial for recognition approaches that instrument frames are first detected in a sequence of video frames for further investigating only these frames. This pre-recognition step is also relevant for many other classification tasks in laparoscopy videos, such as action recognition or adverse event analysis. In this work, the authors address the task of binary classification to recognise video frames as either instrument or non-instrument images. They examine convolutional neural network models to learn the representation of instrument frames in videos and take a closer look at learned activation patterns. For this task, GoogLeNet together with batch normalisation is trained and validated using a publicly available dataset for instrument count classifications. They compared transfer learning with learning from scratch and evaluate on datasets from cholecystectomy and gynaecology. The evaluation shows that fine-tuning a pre-trained model on the instrument and non-instrument images is much faster and more stable in learning than training a model from scratch.

## 1 Introduction

Recording videos and storing them without adding comprehensive notes, that describe these recordings in detail, has become a common practice for many of us and we all know how difficult it is to find a specific scene inside a personal collection of videos. This situation is as well reflected in the field of medical endoscopy, where huge video archives have emerged over the last years that consist of recordings of diverse laparoscopic procedures, stored either for documentation, quality assessment, or training purposes, just to name a few use cases. However, most of these recordings are commonly accompanied by a sparse textual description, which mainly is added to link these stored archives to existing medical case reports. Although videos contain much more information it is impossible to describe everything textually with reasonable effort. Nevertheless, finding specific scenes from such videos archives without a sufficient keyword search is like looking for a needle in a haystack. It is also difficult to describe the content more generally because it has to be considered that some content is relevant for one individual but not relevant for another. Therefore, it becomes apparent that there is a need for making these archives searchable by conducting automatic visual content as well as workflow analysis in order to aid and reduce the workload of medical teams.

In a computer-aided analysis of surgical videos, specifically in examining procedures like cholecystectomy, automated detection of instruments has received attention in many vision-based analysis tasks. For example, automated recognition of surgical workflow steps benefits from the knowledge of which instruments are visible because specific steps are accompanied by specific instruments [1]. However, this issue of detecting instruments is mainly addressed by approaches through classifying images according to their tool presence [2–5], which differs from usual object detection and localisation tasks. This task has recently been studied in the context of automated assessment of surgeons’ performances, which requires information about where instruments are visible because instrument handling is analysed [6]. The detection rate in both tasks, tool presence and spatial localisation alike, has been improved by the usage of deep learning approaches. Nonetheless, there is still room for improvement, and considering the complex task of automatic instrument recognition in laparoscopic videos, this task in its simplest form is to automatically detect instrument frames in a sequence of video frames in order to further investigate solely these frames for recognising different types of instruments.

In this work, we examine the usage of convolutional neural networks (CNNs) in the field of laparoscopy for the purpose of classifying video frames into *instrument* and *non-instrument* frames. Specifically, we address the question to what extent CNNs can be used to classify instrument frames in terms of precision, hit rate, and throughput. Furthermore, we address the question: Which patterns in instrument images are learned and how are they different from patterns in images solely showing tissue? The main contribution of this paper is to review and contrast not only existing approaches towards surgical instrument detection but also state-of-the-art CNN architectures. Although many different network architectures have been proposed since AlexNet [7] was released in 2012, we show that GoogLeNet with batch normalisation is reasonably accurate with sufficient throughput to automatically classify frames in real-time. We validate our findings on two large, independent and publicly available datasets containing different laparoscopic procedures and instrument types. Finally, we assess the classification performance in detail by taking a closer look at activation patterns when processing instrument frames.

## 2 Related work

CNNs have proven to be a highly promising approach for image classification and recognition tasks and undoubtedly, the ImageNet Large Scale Recognition Challenge (ILSVRC) [8] has contributed decisively to advancing networks over the last few years. It has been shown that these approaches achieve considerable performance improvements in terms of accuracy in comparison to traditional approaches, where specific visual features have to be selected manually to describe the task at hand. Among them are AlexNet [7], GoogLeNet [9] alias the Inception network, VGGNet [10] as well as ResNet [11], in the order in which they have participated, and each network has outperformed its predecessor with higher accuracy in the classification task of ImageNet data. Taking a closer look at each aforementioned network according to top-5-accuracy on the ImageNet validation set from ILSVRC 2015, ResNet-152 yields an accuracy of 95.51% (see top-5 error-rate listed in [11] Table 4), whereas VGGNet achieves 92.90%, GoogLeNet 92.11% and BN-InceptionNet even 94.19%.

Extracting visual features to describe instrument appearances have been studied extensively in the last years and the authors of [12–14] investigate various methods to extract such features for specific instrument types appearing in cholecystectomy videos. For examples, Primus *et al.* [13] utilise ORB [15] features, bag-of-features (BoF) and an SVM for classifying different instrument images and obtained a mean average precision (mAP) of $56\percnt \pm 2.0\percnt $ ($n = 6$, see [13] Table I (a) column (512, 16)). Primus *et al.* [14] apply a similar approach but do not process the entire image, they pre-select instrument regions using a histogram-based thresholding method in the CIE L*a*b* chromaticity coordinates and finally process these selections for SVM classifications. Another histogram-based approach is presented by Letouzey *et al.* [12], but in comparison to the previously mentioned work, they apply a greyscale histogram for selecting instrument regions and classify them using HOG features and SVM, resulting in a mAP of 72% ($n = 8$, see total accuracy listed in [12] Table 1). However, results of both approaches are not directly comparable, although similar data and instrument types are investigated – Primus *et al.* [13] use a custom dataset comprising six different instrument classes, whereas Letouzey *et al.* [12] report results based on a dataset, which is part of a challenge at M2CAI 2016 [16].

This tool presence detection challenge at M2CAI 2016 [16] has shown that CNNs are more accurate in classifying entire images according to different instrument types compared to the aforementioned approaches. Zia *et al.* [5] compare the classification performance by fine-tuning pre-trained models of several CNN architectures, which are originally trained on the ImageNet dataset [8]. They achieved an mAP of 63.78% with AlexNet [7], 69.75% with VGGNet [10] and 76.60% with InceptionNet [17] in its third version. In [4], the authors evaluate ToolNet [1], a network based on AlexNet, fine-tune a pre-trained model on the challenging dataset and obtained mAP of $52.5\percnt \pm 30.5\percnt $ ($n = 7$). Another approach taken by Sahu *et al.* [3] is to use off-the-shelf CNN features of a pre-trained model of AlexNet to further train a random forest classifier. This classifier achieves an mAP of $54.50\percnt \pm 24.4\percnt $ ($n = 7$). In addition to [5], Raju *et al.* [2] also fine-tune pre-trained models of VGGNet and GoogLeNet with different setups and report an mAP of 78% using GoogLeNet and 75% for VGGNet. The evaluation of the last two mentioned approaches on the challenging test data has shown that using VGGNet and GoogLeNet are equally accurate as classifying CNN features off-the-shelf [3] and finally both approaches rank at the top.

## 3 Approach

During laparoscopy, surgeons use different instruments for performing different surgical actions. These instruments vary in size and shape and are used either solely or combined with others for actions that require multiple instruments (e.g. suturing). It is common that, for example, gynaecological surgeries are performed with up to three diverse instruments simultaneously. Therefore, we want to find all frames in a surgery video automatically that show laparoscopic instruments of any type, size, or count. To achieve this, we formulate this task as a binary classification task and train CNN to learn the representation of frames that show instruments and the ones that do not. For this task, we select a modified version of GoogLeNet [9] with batch normalisation [18], because this regularises the model with trainable parameters and generalises faster over training data at equal performance as demonstrated by Szegedy *et al.* [17].

*Network architecture:* As described by Szegedy *et al.* [9], GoogLeNet consists of 22 layers, and later Szegedy *et al.* [17] have shown that a batch normalisation function inserted between each of these layers allowing faster and more stable training of a model. However, the batch normalisation technique is evaluated with a modified version of the GoogLeNet, where the main difference of both networks is that a convolutional layer has been replaced by two consecutive smaller convolutional layers. Since the reason for this modification is not stated, we use GoogLeNet in its original version together with batch normalisation. To distinguish between both networks, we take the term ‘*BN-GoogLeNet*’ as by Szegedy *et al.* [17], which represents the batch normalised GoogLeNet in its first version.

**Fig. 1:**
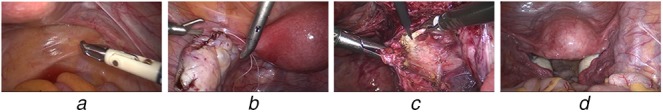
Example images of the instrument count dataset [19], labelled according to the number of visible instruments *a* One *b* Two *c* Three *d* Zero

*Datasets:* For training the model, we use a publicly available dataset that has been published in the context of automatic content analysis in laparoscopic gynaecology by Leibetseder *et al.* [19]. In this Letter, the authors present in total four different datasets, where each of these datasets is built for a specific content analysis problem in the field of laparoscopy. One of them is the automatic determination of the number of visible laparoscopic instruments solely based on processing images, for which they also provide a baseline evaluation. The instrument count dataset consists of images from gynaecology and cholecystectomy (added samples form Cholec80 dataset [1]). In total, the dataset contains ∼22k labelled images, categorised equally into four different classes. Each class represents a specific number of visible instruments, which is shown in Fig. 1: either one instrument (Fig. 1*a*) is visible, or two (Fig. 1*b*), or three (Fig. 1*c*) or there are no instruments visible at all (Fig. 1*d*).

*Data pre-processing:* We process only a subset of the instrument count dataset [19] to learn the classification of instrument and non-instrument images. Since the dataset contains ∼5.1k samples of each instrument count class, we use all images that are labelled with no visible instruments as non-instrument images and a third of every other class as an example for instrument images. This results in a training dataset of 10.2k examples, equally distributed over both classes. To distinguish between both datasets, we introduce the term ‘*InstCnt dataset*’ to describe the entire instrument count dataset of 22k images and ‘*InstBin dataset*’ to denote the subset that is processed for the binary classification task. To detail the image pre-processing settings for training BN-GoogLeNet models, each image and its RGB values are normalised between (–1.0 and 1.0) and squashed to the input image size of the network without cropping. This setup is used for training and validation equally.

## 4 Experiment

In this section, we describe in detail the experimental setup used to train and evaluate BN-GoogLeNet [17] for binary classification of image sets showing laparoscopic instruments. We describe insights obtained on parameter initialisation and analyse the classification performance of several models in terms of precision and hit rate, alias sensitivity. Finally, we test our approach using different types of laparoscopy video data, including video recordings of gynaecology and cholecystectomy. Our experiments are conducted using PyTorch [20] running on Nvidia GeForce GTX TITAN X.

### 4.1 Training and validation

**Fig. 2:**
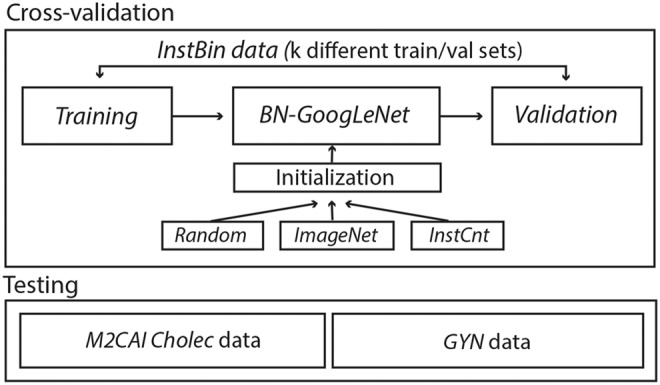
Training phase in a k-fold cross-validation setup with different initialisation methods. Random stands for training from scratch and ImageNet, InstCnt is used as a term where a model is initialised using either a pre-trained ImageNet or InstCnt model. Generalisability is tested on two independent datasets, comprising cholecystectomy (M2CAI Cholec) and gynaecology (GYN) data

We train and validate resulting models with the usage of a k-fold cross-validation approach. In doing so, we select $k = 5$ and split the training dataset with a size of *n* samples into five equal subsets of $n/k$ samples each. Overall, training of the network is carried out five times, where one fold of these subsets is used as validation data with a total of $n/k$ samples and remaining $n - n/k$ samples as training data. In the following, reported results are averaged across each fold unless otherwise stated. We also use the term ‘*training phase*’ for training one fold to distinguish between the averaged values across all folds and evaluation of training on one selected fold. Since we also consider different initialisation approaches to train a BN-GoogLeNet model for the binary classification task, Fig. 2 illustrates a training phase with different setups to initialise a model, where random means a training setup from scratch, ImageNet as well as InsCnt stands for fine-tuning a model either on a pre-trained ‘*ImageNet model*’ or ‘*InstCnt model*’.

*Training the binary classification from scratch:* Initially, a BN-GoogLeNet model is trained on InstBin data from scratch using the Adam [21] optimisation algorithm and the cross-entropy loss function. For optimising the loss function, the algorithm requires the following input parameters: learning rate $\alpha $, stabilisation parameter ${\epsilon}$, decay parameters $\beta _1$, and $\beta _2$. The decay parameters are initialised as suggested in [21] with $\beta _1 = 0.9$ and $\beta _2 = 0.999$, but it is not clear how to choose the learning rate $\alpha $ and the stabilisation parameter $\epsilon$ for our binary classification task. For this parameter, we evaluate several combinations of initialisation and select $\alpha = 10^{ - i}$ and ${\epsilon } = 10^{ - j}$ with *i* out of {1, 2, 3, 4} and *j* out of {0, 1, 2, 3, 4, 8}. The training is conducted for several epochs using a batch size of 64, results in 127 steps per epochs for 8160 training examples. During the hyperparameter search, we found that each combination of ${\epsilon}$ and $\alpha $ results always in an unstable training phase and only a learning rate of $\alpha = 10^{ - 3}$ with ${\epsilon} = 10^{ - 8}$ and $\alpha = 10^{ - 2}$ with ${\epsilon} = 10^{ - 2}$ perform equally well, but the latter combination converges better and faster in terms of validation accuracy.

*Training with transfer learning:* Additionally, we test the initialisation of the BN-GoogLeNet model with a pre-trained model on the ImageNet dataset [8] and a model trained on the InstCnt dataset and uses both models to fine-tune on our data for the binary classification task. For fine-tuning a model on the InstCnt dataset, we re-implement the training setup in [19] and train a model of the network from scratch using the entire InstCnt dataset consisting of 21,433 samples. To perform five-fold cross-validation, we split the dataset into 17,147 samples for training and 4286 samples for validation. Training is conducted as described previously for several epochs using a batch size of 64, results in 267 steps per epoch. Finally, a model for the binary classification task is initialised with both pre-trained models on ImageNet and InstCnt data, respectively. In a fine-tuning setup, all layers of BN-GoogLeNet are re-trained for the new task and only the last fully connected layer is re-initialised for the corresponding number of classes. Since we also use a subset of the instrument count data for fine-tuning, overfitting of the model is an issue, therefore, the model is additionally validated with two independent datasets.

**Fig. 3:**
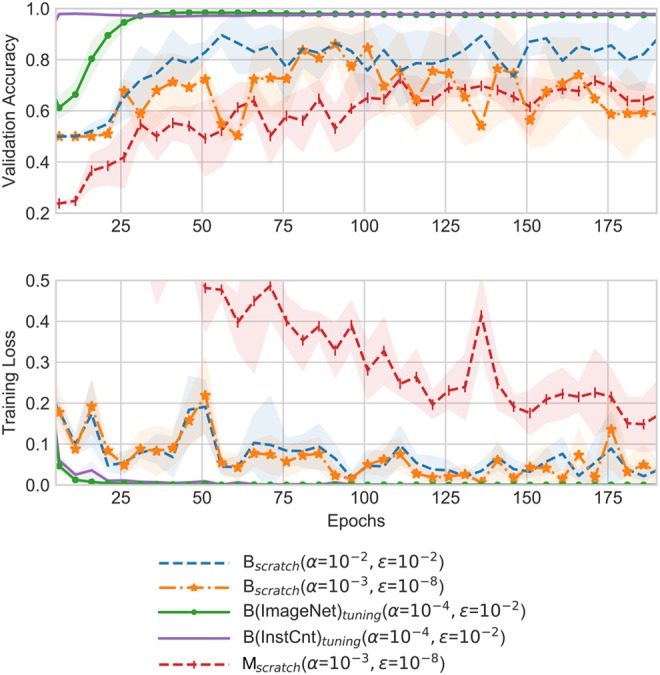
Accuracy and loss during training in five-fold cross-validation averaged over each epoch and line shadows represent standard deviation

*From scratch versus transfer learning:* Fig. 3 shows loss and accuracy in five-fold cross-validation during training the binary classification task *B* (blue and orange dashed lines) and the multi-classification task *M* (red dashed line) from scratch in comparison to fine-tuning a model using ImageNet data (solid green line) and InstCnt data (solid purple line), respectively. Additionally, Table 1 reports the average validation accuracy over five-folds with a standard deviation for the five different training approaches. As shown in Fig. 3, training a model for counting instruments with provided data and suggested default values $\alpha = 10^{ - 3}$, ${\epsilon } = 10^{ - 8}$ results in a slow learning progress and a maximum average accuracy of 67% after epoch 150. Also, higher learning rates $\alpha $ and epsilons ${\epsilon}$ show similar results as with training the binary classification task from scratch but in these cases the validation accuracy decreases after few epochs and does not increase anymore. On the other side, training a model as a binary classifier the same settings of the Adam optimiser as suggested for instrument counts but for a single class (dashed orange line) is much faster and results in more stable training. Also, increasing both parameters to $\alpha = 10^{ - 2}$ and ${\epsilon} = 10^{ - 2}$ (dashed blue line) leads to an improvement of validation accuracy to 88% in epoch 80.

**Table 1:** Overall accuracy of different training setups in five-fold cross-validation at each epoch; the re-implemented setup based on [19] to train a multiclass classifier *M* in comparison to our experimental setup using BN-GoogLeNet to train a binary classifier *B* for different parameters – learning rate $\alpha $ and ${\epsilon}$ of the Adam [21] optimiser. Highest values are bold

Epoch	40	50	60	70	80	90	100	150
Re-implemented [19]								
$M_{\alpha = {10}^{ - 3}\comma {\epsilon} = {10}^{ - 8}}$	0.47 ± 0.04	0.54 ± 0.18	0.59 ± 0.10	0.59 ± 0.08	0.58 ± 0.16	0.58 ± 0.08	0.60 ± 0.12	**0.67** ± 0.06
Ours								
From scratch								
$B_{\alpha = {10}^{ - 3}\comma {\epsilon} = {10}^{ - 8}}$	0.70 ± 0.16	0.72 ± 0.08	0.68 ± 0.19	0.78 ± 0.14	0.62 ± 0.14	**0.83** ± 0.17	0.77 ± 0.16	0.59 ± 0.11
$B_{\alpha = {10}^{ - 2}\comma {\epsilon} = {10}^{ - 2}}$	0.83 ± 0.08	0.87 ± 0.07	0.80 ± 0.14	0.85 ± 0.08	**0.88** ± 0.01	0.82 ± 0.12	0.84 ± 0.09	0.84 ± 0.08
Tuning on ImageNet								
$B_{\alpha = {10}^{ - 4}\comma {\epsilon } = {10}^{ - 2}}$	**0.98** ± 0.01	**0.98** ± 0.00	**0.98** ± 0.00	**0.98** ± 0.00	**0.98** ± 0.00	**0.98** ± 0.00	**0.98** ± 0.00	0.97 ± 0.00
Tuning on InstCnt								
$B_{\alpha = {10}^{ - 4}\comma {\epsilon} = {10}^{ - 2}}$	**0.97** ± 0.01	**0.97** ± 0.01	**0.97** ± 0.01	**0.97** ± 0.01	**0.97** ± 0.01	**0.97** ± 0.01	**0.97** ± 0.01	**0.97** ± 0.01

*Performance details:* Table 2 details the classification performance in training from scratch in terms of precision, sensitivity alias hit rate, and *F*1 score averaged over five-folds. The *F*1 score represents the harmonic mean of precision and sensitivity and is calculated on the basis of

$$F1= 2\ast \displaystyle{{{\rm Precision}\ast {\rm Sensitivity}} \over {{\rm Precision} + {\rm Sensitivity}}}.$$

**Table 2:** Details of classification performance for the best accuracy in training the network from scratch in five-fold cross-validation. Reported results in [19] compared to re-implemented training setup for multi-class (epoch 150) and proposed binary classification (epoch 80)

Classification	Precision	Sensitivity	*F*1
Original [19]			
Zero instruments	0.92	0.93	0.93
One instrument	0.82	0.79	0.80
Two instruments	0.79	0.76	0.77
Three instruments	0.84	0.90	0.95
Re-implemented	** **	** **	** **
${\rm Scratc}{\rm h}_{\alpha = {10}^{ - 3}\comma e = {10}^{ - 8}}$			
Zero instruments	0.76 ± 0.06	0.95 ± 0.02	0.84 ± 0.03
One instrument	0.60 ± 0.07	0.71 ± 0.05	0.65 ± 0.06
Two instruments	0.56 ± 0.07	0.56 ± 0.10	0.56 ± 0.08
Three instruments	0.89 ± 0.04	0.49 ± 0.15	0.61 ± 0.12
Ours			
${\rm Scratc}{\rm h}_{\alpha = {10}^{ - 2}\comma e = {10}^{ - 2}}$			
Non-instrument	0.81 ± 0.02	0.99 ± 0.00	0.89 ± 0.01
Instrument	0.99 ± 0.00	0.76 ± 0.03	0.86 ± 0.02

As can be seen, the results obtained by our re-implemented training setup differ from the results reported in [19]. However, batch normalisation and image pre-processing are reasons why results may differ. It seems that GoogLeNet is trained without batch normalisation since this is not implemented in the provided network version used in [19]. On the other hand, the precision in each class is similar to the original results, where zero and three instrument classifications achieve higher precision compared to other classes. Also, precision is zero (76%) instrument and three (89%) instrument class shows that classification in only two classes is much more promising. The results of the binary classification show that the number of incorrectly classified instrument images as non-instrument images is low because the precision yields a value of 99%. However, this means that instrument images are perfectly classified, but there are many non-instrument images that are classified as an instrument image, as can be seen with a precision of 81% for the non-instrument class. Also, the sensitivity shows that 99% of all non-instrument frames are correctly classified, which means that the classifier classifies more instrument frames as non-instrument frames, which can be attributed to the fact that some instrument frames shows instrument parts that are difficult to learn and distinguishable from non-instrument frames.

### 4.2 Visual explainability

**Fig. 4:**
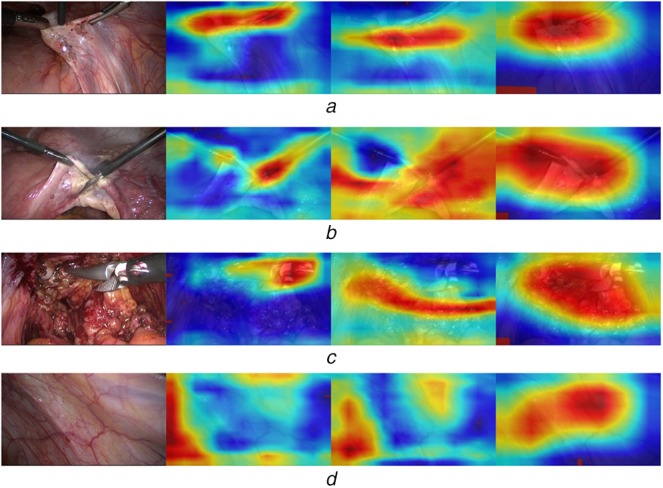
Activation maps of different GoogLeNet layers 4a, 4b, and 5b for a different number of visible instruments. Red regions within the yellow area represent a high activation for the instrument image class in (a–c) and non-instrument image class in (d), whereas the blue colour represents a very weak activation *a* Three instruments – layers (4a, 4b, 5b) *b* Two instruments *c* One instrument *d* Zero instruments

To understand how instrument images are distinguished from non-instrument images, we visualise the regions of the trained BN-GoogLeNet model for the inception layers 4a, 4d and the last layer 5b (listed in Table 1 in [9]). This should help to understand which regions seem to be important for a specific instrument class. We use the gradient-weighted class activation map, described in [22] to visualise the trained weights of different layers for a specific class. Fig. 4 shows the resulting activation maps for instrument and non-instrument images, respectively. The first three images in Figs. 4*a*–*c* represent the activation maps for different number of visible instruments at different layers. The second image of Fig. 4*a* shows the activation of an earlier layer (4a); the third is obtained from a layer in the middle (4d) and the fourth image is from the last layer (5b). As can be seen, instrument regions are localised more precisely in earlier layers and the deeper the image representation is learned, the more coarse-grained the activated weights for this specific class. However, we found that in comparison to instrument images, non-instrument images have no activation patterns in common. Sometimes the entire area leads to high activation, sometimes only small regions in the centre, but also quite often a small region in a corner, which can be seen in the last image (Fig. 4*d*) showing the activation map of the inception layers (4a, 4d and 5b), when processing a non-instrument image.

### 4.3 Throughput

Finally, we measure the throughput in terms of inference time and training time, with frames per second (FPS) on GPU and CPU. In training, we compared the required time in milliseconds to pass a batch of 64 images forward and backward. For inference time, we average the time over 10 runs for 1000 images. As a baseline, we additionally train a model using ResNet [11] on a pre-trained model on ImageNet, which yields a similar maximum accuracy of 98% for the binary classification task of InstBin data.

Results are reported in Table 3, where BN-GoogLeNet yields a high throughput in training and inference with approximately 111 FPS on GPU and 15 FPS on CPU. However, if we compared this throughput to the required time with ResNet [11], measured for two different network sizes, it becomes clear that for a binary classification task BN-GoogLeNet as well as ResNet-50 and ResNet-101 are sufficiently fast to classify frames of laparoscopy videos in real-time on GPU with reasonable accuracy.

**Table 3:** Throughput of different network architectures. Time in milliseconds is measured for one forward and backward pass for one batch of 64 images. Inference time is measured in FPS using GPU and CPU

Model	Forward	Backward	FPS GPU	FPS CPU
BN-GoogLeNet	10.2	16.0	111	15
ResNet-50	15.8	25.3	90	12
ResNet-101	26.1	179.8	47	8

### 4.4 Generalisability

To test the accuracy of classification independently from the training data, we evaluate the performance of the trained model with two further datasets. Among them is a self-annotated dataset of laparoscopic gynaecology videos as well as a publicly available dataset of laparoscopic cholecystectomy videos that are used in the M2CAI challenge [16] for detecting tool presence. For distinguishing between them, we introduce the terms ‘*M2CAI Cholec dataset*’ and ‘*GYN dataset*’.

*Data:* The M2CAI Cholec dataset comprises 15 videos of cholecystectomy procedures, separated into two subsets: a set consisting of 10 videos for training and a test set with five videos. In total, test videos have a duration of 3.65 h and each video is accompanied by annotations indicating the presence or the absence of seven specific laparoscopic instruments. These annotations are provided for one FPS. For the following evaluation, only the test set is used and we process samples as non-instrument images when no label for the presence of any instrument is available and the remaining ones are used as instrument samples. This results in 1880 non-instrument and 10,653 instrument image samples. The second dataset consists of five videos of gynaecologic laparoscopy with a total duration of 5.07 h. Similar to the M2CAI Cholec dataset, one FPS is labelled with instrument or non-instrument, which results in 12,542 instrument samples and 5682 non-instrument samples.

*Classification performance:* Table 4 summarises the results for the InstBin validation data of the training phase. Both classifiers tuned on ImageNet and InstCnt data achieve a maximum precision of 99% on the validation dataset for instrument classifications. The precision for the M2CAI Cholec and GYN data is lower, but it is higher for the GYN data compared to the Cholec data. However, the InstBin dataset comprises images of cholecystectomy and gynaecology, but it seems that the number of images from gynaecology is higher than the number of images from cholecystectomy. Also the classifier tuned on InstCnt data achieves similar results but is less precise in identifying non-instrument images in GYN data and instrument images in M2CAI Cholec data. One reason could be that instruments differ in appearance for cholecystectomy procedures as well as for gynaecologic laparoscopy.

**Table 4:** Classification performance on test datasets using fine-tuning on ImageNet in comparison to InstCnt data at epoch 80 (see Table 1). Precision and *F*1-score are weighted according to the total number of labels in each class. Highest values are bold

Classification	InstBin validation data			M2CAI Cholec data			GYN data		
	Precision_*IB*_	Sensitivity_*IB*_	*F*1_*IB*_	Precision*_CH_*	Sensitivity*_CH_*	F1*_CH_*	Precision*_GY_*	Sensitivity*_GY_*	F1*_GY_*
Tuning on ImageNet									
Non-instrument	**0.99**	**0.99**	0.99	**0.85**	0.82	0.84	0.89	**0.94**	0.92
Instrument	**0.99**	**0.99**	0.99	0.83	**0.86**	0.92	**0.94**	0.89	0.93
Tuning on InstCnt									
Non-instrument	0.97	**1.00**	0.98	**0.96**	**0.86**	0.97	0.77	**0.95**	0.85
Instrument	**0.99**	0.97	0.98	0.85	0.75	0.86	**0.94**	0.72	0.83

**Fig. 5:**
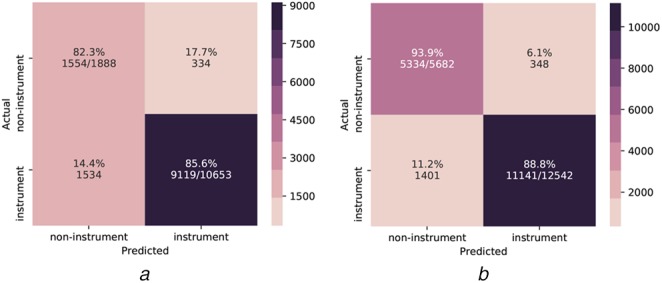
Confusion matrix of the instrument and non-instrument image classifications on two test datasets using fine-tuning on ImageNet, one randomly selected training phase and best performing classifier at epoch 80 *a* M2CAI Cholec dataset *b* GYN dataset

**Fig. 6:**
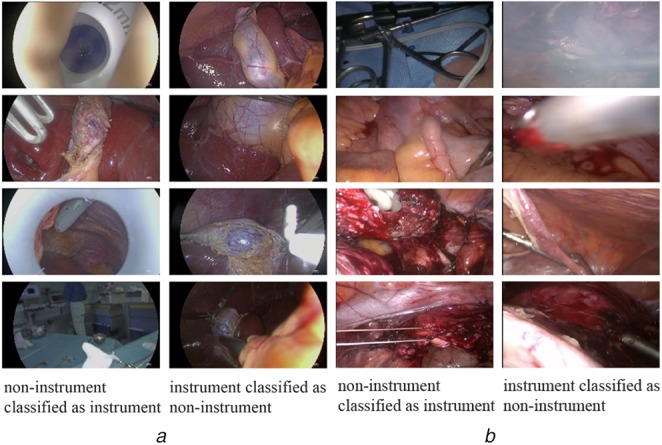
Examples of miss-classifications using fine-tuning on ImageNet, one randomly selected fold and best performing classifier at epoch (80) *a* M2CAI Cholec dataset *b* GYN dataset

*Miss-classification:* For the fine-tuned model on ImageNet data, the confusion matrix of classification results is shown in Fig. 5. Interestingly, more images are classified as instruments in the M2CAI Cholec dataset, although they are labelled as non-instrument images and the opposite is the case for the GYN dataset. Therefore, we take a closer look at miss-classified examples, which are represented in Fig. 6. In both datasets, images that show out-of-patient scenes are wrongly classified as instrument images. The reason could be that such scenes are not included in the InstBin training dataset and are rather classified as instrument images than non-instrument images. We also observe miss-classifications due to heavy smoke, instruments at the corner and strongly deformed appearance of instruments. Furthermore, we notice that many examples in the M2CAI Cholec and the GYN dataset are labelled as non-instrument, although they show at least a piece of an instrument, which explains the lower precision in non-instrument classes.

## 5 Conclusions

In this Letter, we evaluate different approaches for training CNN models in order to identify the instrument and non-instrument images in laparoscopy videos. This is a relevant problem, because it can be used as pre-processing step for any other content classification task in surgery videos such as action recognition or adverse event analysis. In doing so, we use the GoogLeNet [9] in its first version with batch normalisation [18] and train the network with three different initialisation approaches: randomly with training from scratch and transfer learning using two different models for initialisation. For learning from scratch, we use a subset of the publicly available instrument count dataset [19] and compare several initialisation setups of the Adam [21] optimisation algorithm for binary classifying laparoscopy data. For transfer learning, we use two pre-trained models: one model is trained on the ImageNet [8] data and one on the instrument count data (InsCnt). The latter model is obtained by re-implementing the training setup in [19]. Finally, each of these pre-trained models is used to initialise the network while fine-tuning a model for the instrument and non-instrument classification tasks. We compared the classification performance of both approaches using two independent datasets: the M2CAI-tool [1] dataset of cholecystectomy procedures (M2CAI Cholec data) and a self-annotated dataset of gynaecology procedures (GYN data).

In our experiments, we found that learning the binary classification task of the instrument and non-instrument images from scratch is unstable for several initialisation setups of the Adam [21] optimiser and an optimal parameter configuration could not be determined. However, fine-tuning a network model for this classification task results in a faster and more stable training and the classification performance is more accurate ($98\percnt \pm 0\percnt $) than training from scratch ($88\percnt \pm 1\percnt $), evaluated in five-fold cross-validation. Also, the classification performance on two independent test datasets indicates that instrument images can be identified with 94% precision in gynaecology procedures. However, the non-instrument class yields a lower precision because out-of-patient images are often classified as instrument images, while labelled as non-instrument images. Therefore, we additionally investigate in this Letter which regions are activated at different inception layers for instrument and non-instrument frames, respectively. We found that instrument frames have in common similar activation patterns and these patterns reflect instrument regions, whereas non-instrument frames have arbitrary activated regions and it seems that the tissue does not lead to these activations. This could be one reason why frames with unusual structures like out-of-patient images are rather classified as instrument frame than as non-instrument frame.

Finally, the most important findings are twofold: (i) simple network architectures for simple classification tasks achieve similarly accurate results but with much higher throughput and (ii) even though application domains may strongly differ, it can be worthwhile to consider transfer learning for specific domain classification tasks, while additionally having the advantage of faster converging models.

## References

[1] Twinanda A.P. Shehata S. Mutter D.: ‘Endonet: a deep architecture for recognition tasks on laparoscopic videos’, IEEE Trans. Med. Imaging, 2016b, 360, (1), pp. 86–97 (doi: 10.1109/TMI.2016.2593957) 10.1109/TMI.2016.2593957 27455522

[2] Raju A. Wang S. Huang J.: ‘M2CAI surgical tool detection challenge report’. Workshop and Challenges on Modeling and Monitoring of Computer Assisted Intervention (M2CAI), Athens, Greece, Technical report, 2016 Available at http://camma.u-strasbg.fr/m2cai2016/reports/Raju-Tool.pdf

[3] Sahu M. Mukhopadhyay A. Szengel A.: ‘Tool and phase recognition using contextual CNN features’. Workshop and Challenges on Modeling and Monitoring of Computer Assisted Intervention (M2CAI), Athens, Greece, Technical report, 2016 Available at http://camma.u-strasbg.fr/m2cai2016/reports/Sahu-ToolandWorkflow.pdf

[4] Twinanda A.P. Mutter D. Marescaux J.: ‘Single- and multi-task architectures for tool presence detection challenge at M2CAI 2016’. Workshop and Challenges on Modeling and Monitoring of Computer Assisted Intervention (M2CAI), Athens, Greece, Technical report, 2016a Available at http://camma.u-strasbg.fr/m2cai2016/reports/Twinanda-Tool.pdf

[5] Zia A. Castro D. Essa I.: ‘Fine-tuning deep architectures for surgical tool detection’. Workshop and Challenges on Modeling and Monitoring of Computer Assisted Intervention (M2CAI), Athens, Greece, Technical report, 2016 Available at http://camma.u-strasbg.fr/m2cai2016/reports/Zia-Tool.pdf

[6] Jin A. Yeung S. Jopling J.: ‘Tool detection and operative skill assessment in surgical videos using region-based convolutional neural networks’. IEEE Winter Conf. on Applications of Computer Vision (WACV), Lake Tahoe, NV, USA, 2018, pp. 691–699

[7] Krizhevsky A. Sutskever I. Hinton G.E.: ‘Imagenet classification with deep convolutional neural networks’. Proc. of the 25th Int. Conf. on Neural Information Processing Systems (NIPS), Lake Tahoe, Nevada, 2012, pp. 1097–1105

[8] Russakovsky O. Deng J. Su H.: ‘Imagenet large scale visual recognition challenge’, Int. J. Comput. Vis., 2015, 1150, (3), pp. 211–252 (doi: 10.1007/s11263-015-0816-y)

[9] Szegedy C. Liu W. Jia Y.: ‘Going deeper with convolutions’. Proc. of the IEEE Computer Society Conf. on Computer Vision and Pattern Recognition, Boston, MA, USA, 2015, vol. 07-12-June, pp. 1–9

[10] Simonyan K. Zisserman A.: ‘Very deep convolutional networks for large-scale image recognition’. Int. Conf. on Learning Representations, Vancouver, BC, Canada, 2015, p. 14

[11] He K. Zhang X. Ren S.: ‘Deep residual learning for image recognition’. IEEE Conf. on Computer Vision and Pattern Recognition (CVPR), Las Vegas, NV, USA, 2016, pp. 770–778

[12] Letouzey A. Decrouez M. Agustinos A.: ‘Instruments localisation and identification for laparoscopic surgeries’. Workshop and Challenges on Modeling and Monitoring of Computer Assisted Intervention (M2CAI), Athens, Greece, Technical report, 2016 Available at http://camma.u-strasbg.fr/m2cai2016/reports/Letouzey-Tool.pdf

[13] Primus M.J. Schoeffmann K. Böszörmenyi L.: ‘Instrument classification in laparoscopic videos’. Proc. of the Int. Workshop on Content-Based Multimedia Indexing (CBMI), Prague, Czech Republic, 2015, vol. 2015-July, pp. 1–6

[14] Primus M.J. Schoeffmann K. Böszörmenyi L.: ‘Temporal segmentation of laparoscopic videos into surgical phases’. Proc. of the Int. Workshop on Content-Based Multimedia Indexing (CBMI), Bucharest, Romania, 2016, vol. 2016-June, pp. 1–6

[15] Rublee E. Rabaud V. Konolige K.: ‘ORB: an efficient alternative to SIFT or SURF’. Proc. of the IEEE Int. Conf. on Computer Vision (ICCV), Barcelona, Spain, 2011, pp. 2564–2571

[16] M2CAI Challenge. Tool Presence Detection Challenge Results, 2016. Workshop and Challenges on Modeling and Monitoring of Computer Assisted Interventions. Available at http://camma.u-strasbg.fr/m2cai2016/index.php/tool-presence-detection-challenge-results

[17] Szegedy C. Vanhoucke V. Ioffe S.: ‘Rethinking the inception architecture for computer vision’. IEEE Conf. on Computer Vision and Pattern Recognition (CVPR), Las Vegas, NV, USA, 2016, pp. 2818–2826

[18] Ioffe S. Szegedy C.: ‘Batch normalization: accelerating deep network training by reducing internal covariate shift’. 32nd Int. Conf. on Machine Learning, Lille, France, 2015, vol. 37, p. 9

[19] Leibetseder A. Petscharnig S. Primus M.J.: ‘Lapgyn4: a dataset for 4 automatic content analysis problems in the domain of laparoscopic gynecology’. Proc. of the 9th ACM Multimedia Systems Conf. on (MMSys), New York, New York, USA, 2018, pp. 357–362

[20] Paszke A. Gross S. Chintala S.: ‘Automatic differentiation in PyTorch’. NIPS Autodiff Workshop, Long Beach, CA, USA, 2017, pp. 1–4

[21] Kingma D.P. Ba J.: ‘Adam: a method for stochastic optimization’. Int. Conf. on Learning Representations (ICLR), Banff, AB, Canada, 2014, p. 13

[22] Selvaraju R.R. Cogswell M. Das A.: ‘Grad-CAM: visual explanations from deep networks via gradient-based localization’. Proc. of the IEEE Int. Conf. on Computer Vision (ICCV), Venice, Italy, 2017, pp. 618–626

